# Editorial: New insights into sleep abnormalities associated with alcohol, cannabis, cocaine, and opiate use

**DOI:** 10.3389/fpsyt.2024.1481765

**Published:** 2024-10-21

**Authors:** Tatiana Ramey

**Affiliations:** National Institute on Drug Abuse (NIDA), Bethesda, MD, United States

**Keywords:** sleep abnormalities, substance use, alcohol, cannabis, cocaine

Sleep abnormalities are known to relate to substance use in multiple ways and levels, connected via underlying common neurobiological mechanisms. Sleep-related problems feature prominently in clinical presentations of substance withdrawal, craving, and relapse, and are associated with poor treatment outcomes. Substance use alters circadian rhythms, negatively affecting the sleep-wake cycle, thus creating a vicious circle where drug use and sleep impairment reinforce each other. Additionally, people with substance use disorders (SUD) continue to commonly have sleep abnormalities after cessation of non-prescribed substance use, with these sleep problems persisting into SUD treatment and recovery. Treatment of sleep problems therefore could positively impact substance use outcomes, improving withdrawal symptoms, preventing relapse, and, albeit indirectly, decreasing instances of drug overdose.

Our Research Topic has gathered a mix of insights into sleep disturbances, their current treatments, and substance use, such as alcohol, cannabis, cocaine, and opiates.

Soyka et al. work brings to light the current European situation that is somewhat paradoxical when treatment for insomnia tends to be benzodiazepines and related z-drugs, such as eszopiclone, zolpidem, and zaleplon. There are multiple risks in using these medications, especially chronically. Most importantly, the use of benzodiazepines and related z-drugs to treat insomnia simultaneously increases the risk of tolerance and benzodiazepine dependence, other drug misuse and dependence, and polysubstance use. Therefore, non-benzodiazepine compounds are needed to treat people with sleep problems, while current benzodiazepine drugs are inadequate for this purpose.

Another insight is added by the mixed-methods study of Eglovitch et al. that investigates sleep and its connection with opioid use disorder (OUD) recovery in patients stabilized on buprenorphine for OUD treatment. Little is known about patient’s preferences in how their sleep disturbances are treated, especially women. This study took women participants from an ongoing cross-sectional survey to complete a qualitative interview. Importantly it came out that insomnia medications were perceived as carrying a risk of dependence, and patients indicated that they would rather tolerate poor sleep than take a “sleeping pill”. Related to a gendered factor among this woman sample, it was revealed that caregiving was seen as a barrier to receiving insomnia treatment, and the sedating effect of sleep medications was seen as negatively affecting their caregiver function. Aside from women’s societal pressures and roles in caregiving and family, social determinants of health (transportation barriers, internet and phone connectivity) were discovered to affect both pharmacological and non-pharmacological treatments, for both in-person and telehealth visits.

Milanak et al. demonstrate to us how a transdiagnostic approach might work in addressing sleep disturbances and anxiety symptoms in a polysubstance use and comorbid population. They investigated the feasibility and preliminary effectiveness in a single-arm pilot trial of an empirically informed group transdiagnostic intervention, Transdiagnostic SUD Therapy, to concurrently reduce anxiety and improve sleep among adults receiving intensive outpatient treatment for SUD. This behavioral treatment of sleep and anxiety was used in a group of subjects irrespective of the specific substance used and with comorbidities. The study sample was comprised of 163 participants with various SUDs, and about a third of the study population also had comorbid depression and anxiety. Transdiagnostic SUD group therapy sessions were specifically designed to be feasible in real-world intensive outpatient program settings. The therapy itself represented a CBT protocol with sleep and anxiety modules. Components of the sleep modules included sleep education, sleep restriction, stimulus control, values clarification and values-consistent behavioral activation, cognitive restructuring, and sleep hygiene. Anxiety modules included psychoeducation, worry time, interpersonal effectiveness skills, mindfulness and acceptance, and relaxation strategies. Overall, a significant decrease in anxiety and insomnia severity as well as a sleep improvement were demonstrated as a result of the 4-week duration group therapy, albeit under open-label conditions. This work underscores the importance and value of a transdiagnostic approach to behavioral treatment in SUD, especially where real-world applicability was considered.

Shan et al. carried out a case-control study among a sample of adult males undergoing scheduled orthopedic surgery. The sample consisted of 93 non-smoking and 53 participants who smoked at least 10 cigarettes a day. All participants underwent a lumbar puncture, and cerebrospinal fluid samples were analyzed for insulin-like growth factor-1 (IGF1). In prior research, both levels of IGF1 and nicotine use have demonstrated associations with sleep quality. Thus, this study explored the moderating effect of IGF1 on the association between smoking cigarettes with scores on the Pittsburgh Sleep Quality Index, reporting a significantly positive moderation. These findings, the authors state, bring new insights into the intersection of tobacco use and sleep quality, in particular illustrating IGF1 as a potential target for new insomnia interventions among people with nicotine use disorder.

The last article in our grouping is by An et al. - that is where insights on sleep disturbances are gained from a different angle - a cross-sectional study using data from the National Health and Nutrition Examination Survey (NHANES) from 2005 to 2006 and 2007 to 2008 cycles. This study concentrated on the association between folic acid serum levels and difficulty falling asleep, –defined as reporting over 15 times difficulty falling asleep situations per month. All participants’ data was subdivided into two groups: the severe difficulty falling asleep group and those not meeting those criteria were treated as a control group. Multivariable logistic regression models revealed a correlation that high levels of serum folic acid were significantly related to fewer odds of severe difficulty in falling asleep among US adults in general, suggesting that folic acid supplementation may be beneficial, potentially in adults with alcohol use disorder where folic acid depletion is common. The authors discuss that, among other hypotheses, it is plausible that altered serum folic acid levels might modulate circadian functioning, thereby impacting sleep quality.

In conclusion, the papers included in this Research Topic offer a mix of insights into sleep abnormalities that accompany substance use disorders. More research in the area of circadian rhythm, sleep abnormalities, and substance use is warranted, as the prevention and treatment of disturbed sleep, being a major factor in all of the facets of alcohol and drug use could lead to improvement of clinical outcomes in substance use disorders.

